# Experimental Investigation of Laser Parameters Dependence of Surface Graphitization in Nanosecond Laser Ablation of  Nanocrystalline Diamond

**DOI:** 10.3390/mi16040374

**Published:** 2025-03-26

**Authors:** Huixin Yuan, Chunyu Zhang, Chengwei Song, Zhibing He, Guo Li, Leyao Li

**Affiliations:** Laser Fusion Research Center, China Academy of Engineering Physics, Mianyang 621900, China; hithuixin@163.com (H.Y.);

**Keywords:** nanocrystalline diamond, graphite, laser ablation, graphitization, nanosecond pulsed laser

## Abstract

Nanocrystalline diamond (NCD) is regarded as a highly promising composite engineering material owing to its superior mechanical properties. Surface texturing significantly enhances the surface performance of NCD. Given the unique inherent combination of hardness and brittleness in NCD, laser ablation emerges as a critical method for fabricating surface microstructures. However, the research on laser-induced surface texturing of NCD remains limited. This study experimentally investigated the characteristics of nanosecond laser-ablation-induced graphitization in NCD and provided an in-depth analysis of the laser ablation mechanism, aiming to guide the optimization of NCD surface microtexture manufacturing. Specifically, we conducted systematic nanosecond pulse laser ablation experiments on NCD samples and utilized Raman spectroscopy to qualitatively characterize the graphitization within microgrooves and across the entire ablated surface. The effects of the laser scanning speed, power, defocus level, and scanning interval on the graphitization extent and morphological characteristics were systematically investigated, identifying the single-factor optimal parameter set for maximizing graphitization. Through single-factor experimental analysis, the findings of this study provide foundational data for subsequent multivariate-coupled optimization and offer theoretical support for enhancing the surface properties of NCD through microtexturing via laser ablation.

## 1. Introduction

Nanocrystalline diamond (NCD) is an advanced engineering material that has attracted significant attention owing to its superior physical and chemical properties [1,2,3,4]. Comprising nanoscale diamond grains, NCD exhibits hardness and elastic modulus values that are comparable to those of single-crystal diamond [5,6,7,8]. Additionally, it possesses ultra-high hardness, high thermal conductivity, a low friction coefficient, and excellent chemical inertness, which render it highly suitable for applications in coatings and tool materials. The introduction of textured surface structures can further enhance its surface properties, such as improving its wear resistance and self-lubrication, thereby offering substantial benefits in the fields of tribology and high-precision machining [7,9,10,11,12,13].

Laser surface texturing technology represents an advanced surface modification technique that significantly enhances the performance of diamond and related materials in various fields, including cutting, tribology, biomedicine, and optics. This technology has garnered considerable attention and is now widely employed in research involving diamond materials. Calvani et al. utilized femtosecond lasers to create nanoscale textures on chemical vapor deposition (CVD) diamond surfaces, investigating their impact on infrared absorption properties. Their findings revealed that laser texturing markedly improves infrared absorption, with the texture depth and width playing crucial roles in this enhancement. Specifically, the absorption rate peaked when the texture depth reached 100 nm [14]. Hazzan et al. focused on controlling the microtexture morphology of diamond surfaces through adjustments in the laser pulse energy density and pulse width. The results demonstrated that at a pulse energy of 50 μJ and repetition frequency of 1 kHz, approximately 2 μm deep pits were formed on the diamond surface; lower-energy pulses (25 μJ) yielded shallower textures [15]. Tan et al. further explored the use of acoustic emission (AE) technology to monitor the laser microtexturing process of polycrystalline diamond (PCD) tools in real-time. The acoustic emission signals provided insights into the surface changes during laser ablation. For instance, as the laser pulse energy increased to 100 mJ, the texture depth reached 2 μm, and a peak AE signal was detected within the 300 kHz frequency range, confirming the significant influence of laser ablation on material properties [16]. Mastellone et al. applied ultra-short pulse lasers to achieve nanoscale texturing on boron-doped diamond films, examining the effects of the laser energy density on the surface morphology. At an energy density of 0.8 J/cm^2^, uniform nanoscale textures, approximately 50 nm deep, were formed. As the energy density increased, the texture depth deepened, accompanied by the formation of pores that were up to 100 nm in diameter [17]. Despite these advancements, systematic studies on laser surface texturing of nanopolycrystalline diamond remain limited, necessitating further investigation into the underlying mechanisms and performance optimization.

Under laser ablation, diamond undergoes graphitization, forming conductive graphite layers. This process provides a viable technical approach for the development of high-performance conductive components. The graphitization phase transition involves the transformation of carbon atoms from an sp^3^-hybridized diamond structure to an sp^2^-hybridized graphite structure under laser ablation [18,19]. Feudis et al. demonstrated that irradiating diamond with high-energy laser pulses resulted in the formation of high-quality graphite layers on its surface, enhancing the electrical conductivity by nearly 30-fold and rendering it suitable for high-performance electrode materials [20]. Zhang et al. employed infrared nanosecond laser ablation on single-crystal diamond, revealing the formation of both a thermally induced transformation layer and a deposited transformation layer. The former serves as a transitional layer from diamond to graphite, while the latter consists of graphite and amorphous carbon, formed through mechanisms including carbon island nucleation and deposition [21]. Odake et al. compared the effects of ultraviolet nanosecond laser ablation on single-crystal and polycrystalline diamond. They observed that the interface between single-crystal diamond and the graphite layer exhibited slight undulations, whereas the interface of the polycrystalline diamond was more linear, with an approximately 1 μm thick graphite layer covering the groove surface [22]. Ohfuji et al. investigated the fabrication of microgroove structures on single-crystal diamond using ultraviolet nanosecond lasers, resulting in graphite layers composed of single-crystal, polycrystalline, and nanocrystalline graphite [23]. However, research on the graphitization of NCD under laser ablation remains limited. Due to its unique nanocrystalline grain structure and high grain boundary density, the graphitization behavior of NCD differs significantly from that of single-crystal and CVD diamond.

The laser ablation parameters significantly influence the graphitization process. Feudis et al. observed that at a laser pulse energy of 60 μJ, the diamond surface initiates graphitization, leading to a tenfold increase in electrical conductivity. The depth and extent of graphitization were found to increase with higher energy levels, which holds potential implications for sensor applications [24]. Ali et al. demonstrated that significant graphitization occurs at an energy density of 2 J/cm^2^ using 30 fs ultra-short laser pulses on diamond, with the graphitization depth being positively correlated with the number of laser pulses [25]. Kononenko et al. reported that diamond gradually undergoes graphitization at an energy density of 1.5 J/cm^2^ through 1 ns laser pulses, while high energy densities result in surface cracking and damage [26]. However, there is currently limited research on the effects of laser ablation on NCD.

In this study, an experimental investigation was conducted on the laser ablation of NCD. The primary objective was to examine the graphitization phenomenon induced by laser ablation and its correlation with various laser ablation parameters. Through systematic experimentation, optimized laser ablation parameters were identified, which enabled the complete surface graphitization of NCD.

## 2. Materials and Methodologies

The NCD samples in this study were synthesized via hot-filament chemical vapor deposition (HF-CVD) with diamond-pellet substrate pretreatment to optimize the nucleation density, following standardized heteroepitaxial protocols for NCD fabrication [27]. The resulting NCD sample measured 2 mm × 2 mm × 1 mm.

**Figure 1:**
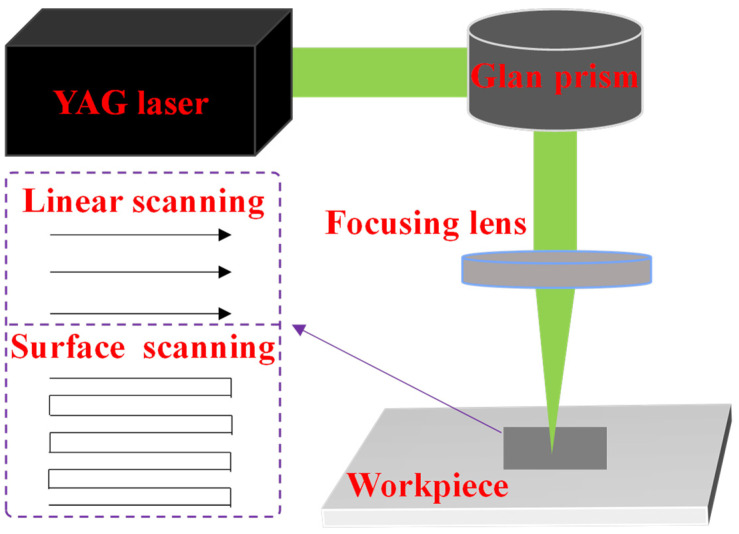
Illustration of the experimental configuration of nanosecond pulse laser ablation.

Figure 1 illustrates the experimental setup of the nanosecond laser ablation of NCD. Laser linear scanning refers to the one-dimensional movement of the laser beam along a predefined trajectory, generating continuous linear coverage. Surface scanning is achieved through periodic reciprocating linear scans with orthogonal directional progression, enabling complete coverage of the target two-dimensional plane. The experimental setup employed a nanosecond pulse YAG laser as the light source, emitting green light at a wavelength of 532 nm, with a pulse duration of 5 ns and a repetition rate of 1 kHz. Essential components of the apparatus for the laser-ablated graphitization of diamond comprised various lenses, the laser system, and the workpiece. The laser system functioned as the irradiation source, whereas the Glan prism was utilized to accurately regulate the laser power output, achieving a precision of 1 mW. The laser ablation process was divided into two types: laser linear scanning and laser surface scanning.

As detailed in Table 1, the laser power was varied from 15 to 35 mW at 5 mW intervals. Five scanning speeds (0.05–0.25 mm/s at 0.05 mm/s intervals) and five defocus levels (0–0.4 mm at 0.1 mm intervals) were combined to investigate their effects on the surface morphology. The scanning intervals were adjusted from 0 to 12 μm with 2 μm increments, with each parameter set being tested in triplicate to ensure statistical significance.

The surface morphology of the NCD specimens after laser ablation was examined using a Scanning Electron Microscope (SEM) with a 3 nm resolution and a sample size capacity of 200 mm × 200 mm × 35 mm. The SEM’s magnification range from X5 to X300,000 enables detailed, high-resolution observation of laser-ablated surface modifications. The structural transformation of NCD under laser irradiation was systematically investigated through Raman spectroscopy, a critical analytical technique for probing carbon phase transitions. The spectral characterization was performed utilizing a 532 nm excitation source, selected for its characteristic high scattering cross-section in diamond matrices. This wavelength demonstrates particular efficacy in resonantly exciting sp^3^-bonded carbon vibrational modes while maintaining structural integrity through reduced photon energy deposition. The optimized excitation parameters enable enhanced detection sensitivity through improved signal-to-noise ratios, coupled with the minimized photodegradation risks that are inherent to high-power laser–matter interactions. The Raman spectrum of NCD exhibits characteristic peaks at 1332 cm^−1^ (diamond peak) and 1350 cm^−1^ (D peak), along with a G peak at ~1580 cm^−1^. The G peak corresponds to the in-plane vibration of the sp^2^-hybridized carbon in graphitic domains, whereas the D peak arises from structural disorder or defects. The absolute I_G_/I_D_ intensity ratio was employed to quantify the graphitization degree. The I_D_/I_G_ ratio was determined from the integrated intensity ratio of D and G bands using Gaussian curve fitting. The Gaussian profiles were selected due to the amorphous nature of laser-induced carbonization products, where structural disorder induces asymmetric peak broadening through phonon localization effects, as well established in the Raman analysis of disordered carbons [28]. The analysis in Origin incorporated adaptive baseline correction via penalized least squares and constrained band fitting, ensuring physically meaningful parameterization. A higher degree of graphitization correlates with a greater I_G_/I_D_ ratio. This correlation arises because ‘I_G_’ represents the peak intensity of graphitized carbon, whereas ‘I_D_’ indicates the peak intensity of amorphous carbon. An elevated I_G_/I_D_ ratio reflects a reduced proportion of amorphous carbon relative to graphitized carbon, thus signifying an improvement in the extent of graphitization.

## 3. Results and Discussion

### 3.1. The Effect of the Laser Scanning Speed on the Graphitization of NCD

**Figure 2:**
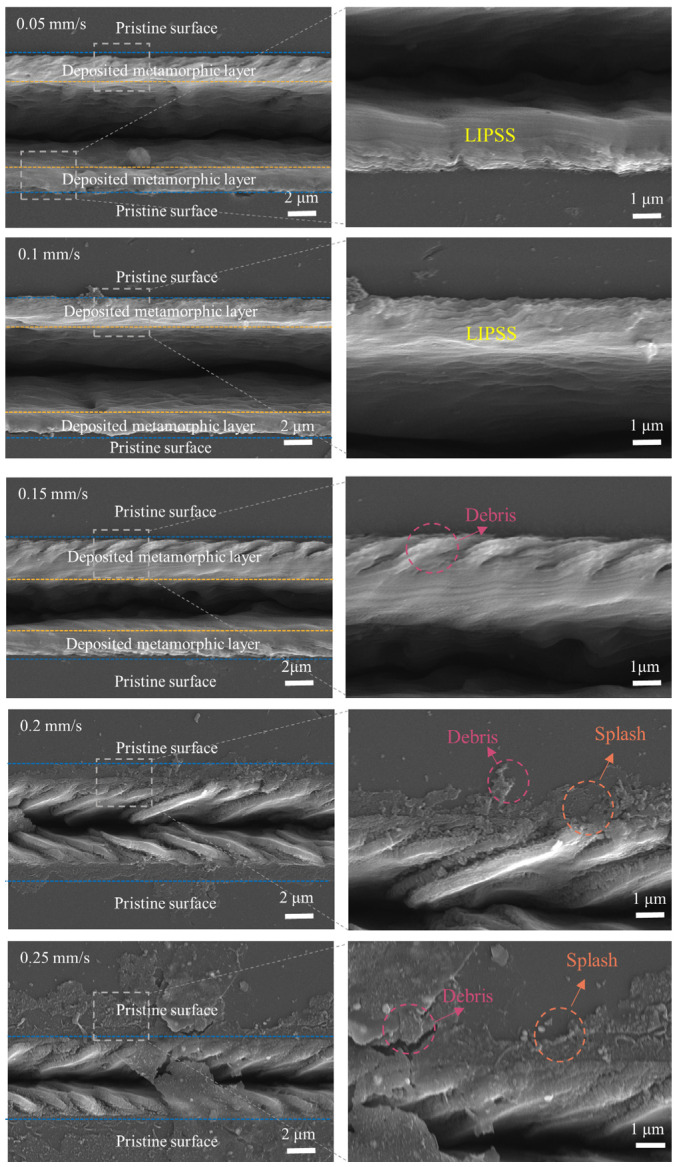
The influence of the laser scanning speed on the morphology of the ablated NCD surface.

As established in our prior study [29], the laser parameters for spot-scan mode were optimized for single-point graphitization of nanocrystalline diamond, where static energy deposition suits precise micro-hole fabrication. The transition to line scan mode necessitates the re-evaluation of parameters due to continuous thermal accumulation during dynamic scanning. Specifically, the coupling effect between the laser power and scanning velocity critically influences the energy gradient distribution in graphitized regions. Nanosecond laser ablation of NCD was performed to investigate the influence of the laser scanning speed on the graphitization of NCD. Six laser scanning speeds, namely 0.05 mm/s, 0.1 mm/s, 0.15 mm/s, 0.2 mm/s, and 0.25 mm/s, were considered. For each laser scanning speed, the other laser ablation parameters were the same, with a laser power of 25 mW and a defocus level of 0 mm. Figure 2 shows SEM images of the ablated surface of NCD under different pulse repetition rates. It can be observed that initially, at lower scanning speeds (0.05, 0.1, and 0.15 mm/s), distinct deposit layers are formed on the surface. This is because the temperature of the NCD surface rapidly increases under the laser ablation. When the temperature at the laser beam’s irradiation position is sufficiently high, a certain degree of evaporation and gasification occurs on the NCD surface, generating gasification products. These products redeposit on the processing surface, forming the deposit layers. Additionally, long-period surface structures (LIPSSs) emerge within the deposit layers. This might be attributed to the longer residence time of the laser beam on the material surface at these lower scanning speeds, resulting in a significant enhancement of local heat accumulation. Meanwhile, due to the periodicity of the laser ablation, the surface forms LIPSSs with periodic characteristics [30,31]. Simultaneously, at a scanning speed of 0.15 mm/s, uniformly distributed fragments appear at the edge of the deposit layer, indicating that during the processing, the local thermal effect of the laser beam might cause the fracture and generation of microstructures on the NCD surface. The occurrence of such fragments may be closely related to factors such as the laser power, local melting, and the cooling rate. The local thermal effect of the laser may cause a concentration of stress on the NCD surface, thereby inducing local fractures of the surface structure and the formation of fragments.

When the scanning speed is further increased to 0.2 mm/s, the deposit layer no longer appears conspicuously. A higher scanning speed implies that the laser beam moves faster on the surface, and the NCD surface can absorb relatively less laser energy, preventing the NCD surface from reaching a sufficient temperature to cause significant deposition of gasification products. When the scanning speed is further increased to 0.25 mm/s, obvious splattering phenomena occur on the surface. This might be because the laser beam heats the local area to an extremely high temperature within a short period, generating excessive vapor or gas and thereby causing the splattering of molten diamond. Furthermore, at scanning speeds of 0.2 and 0.25 mm/s, discontinuous phenomena appear within the surface microgrooves, suggesting that the further increase in the laser scanning speed makes the surface processing more complex. Under the condition of high-speed scanning, it may not be able to effectively carry out local melting and gasification, resulting in discontinuity of the inner surface of the microgrooves.

**Figure 3:**
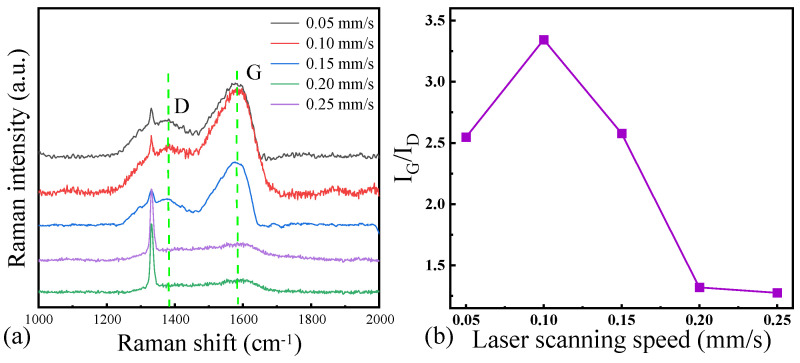
(**a**) Raman spectra and (**b**) l_G_/l_D_ ratio of the surface of NCD at different laser scanning speeds.

Figure 3a shows the results of the Raman spectroscopy on the surface of the laser-ablated microgrooves at different laser scanning speeds, and Figure 3b shows the corresponding I_G_/I_D_ ratio in the Raman spectroscopy curve. The integrated intensities at the intersection points between the green lines of the D and the G peak and the Raman curve were adopted as the I_D_ and I_G_ values, respectively. The observations of the G peak and the D peak indicate varying degrees of graphitization on the NCD surface during laser ablation. Initially, when the laser power was 25 mW and using the six laser scanning speeds, namely 0.05 mm/s, 0.1 mm/s, 0.15 mm/s, 0.2 mm/s, and 0.25 mm/s, distinct D and G peaks emerged in the surface Raman spectra, signifying that partial graphitization occurred on the NCD surface under these laser ablation conditions. Specifically, at speeds of 0.05 mm/s, 0.1 mm/s, and 0.15 mm/s, the signals of the D and G peaks were relatively intense, indicating the existence of a certain graphitized layer on the surface. The non-monotonic dependence of graphitization on the scanning speed originates from competing mechanisms: At low speeds (0.05 mm/s), extended laser exposure induces excessive thermal evaporation of graphitic phases, suppressing surface graphitization. Moderate speeds (0.1 mm/s) achieve an optimized balance—sufficient to activate phase transformation while minimizing ablation loss. Further speed increases (>0.1 mm/s) reduce the energy deposition below the critical level required for sustained structural evolution. Particularly at scanning speeds of 0.2 mm/s and 0.25 mm/s, the D band intensity decreases significantly to near the detection limit, and the reduced signal-to-noise ratio may introduce uncertainties in peak integration. Nevertheless, the decreasing trend of the I_G_/I_D_ ratio with increasing scanning speed suggests a reduction in graphitization. Higher scanning speeds and powers might lead to local overheating on the NCD surface, resulting in an uneven deposition layer and the generation of more carbon fragments. These fragments did not undergo a sufficient graphitization process, thereby causing the ratio of the D and G peaks in the Raman spectra to decrease. Through the combination of the surface organization analysis of the deposition layer, at a scanning speed of 0.1 mm/s, the surface deposition layer was relatively smooth and uniform with no obvious fragments, suggesting that the degree of graphitization on the NCD surface was the highest under this condition. Lower scanning speeds and moderate laser powers enabled the surface to maintain a relatively uniform temperature distribution, thereby promoting the graphitization process and forming a smooth and structurally uniform graphitized layer.

### 3.2. The Effect of the Laser Power on the Graphitization of NCD

**Figure 4:**
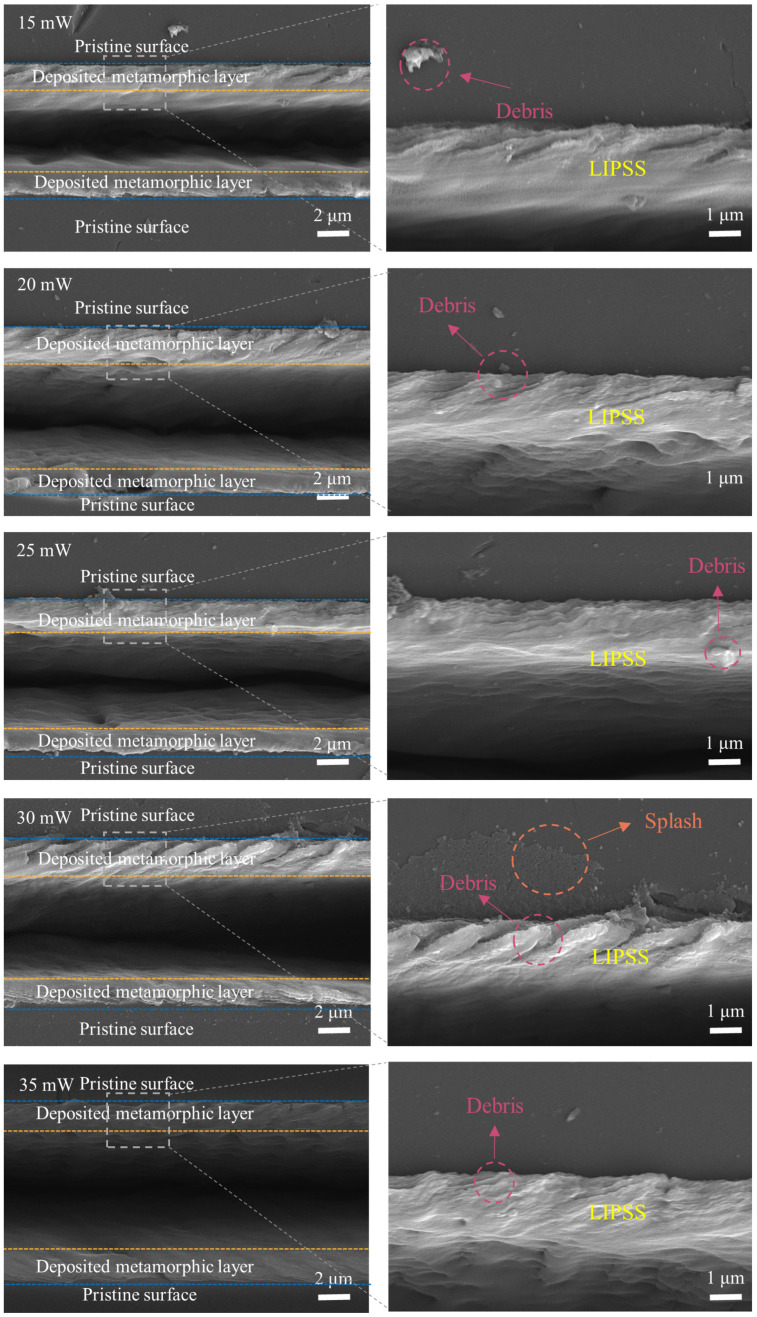
The influence of the laser power on the morphology of the ablated NCD surface.

With the optimal laser scanning speed of 0.1 mm/s, subsequent nanosecond laser ablation of NCD was carried out to investigate the effect of the laser power on the graphitization of NCD. The laser power ranged from 15 mW to 35 mW, with an interval of 5 mW. For each laser power, the other laser ablation parameters were the same, using a laser scanning speed of 0.1 mm/s and a defocus level of 0 mm. Figure 4 illustrates the ablated surface morphology of the NCD at varying laser powers. These morphologies can be categorized into three primary regions as microgrooves, deposited zones, and the diamond matrix, respectively. Firstly, with the increase in laser power, the width of the microgroove gradually expands. This phenomenon can be attributed to the relationship between the laser power and material removal efficiency. Lower powers (such as 15 mW and 20 mW) are insufficient to supply adequate thermal energy to fully melt and remove the material on the NCD surface, while higher powers (such as 30 mW and 35 mW) provide more energy, resulting in the melting and removal of more diamond material and thereby increasing the width of the microgroove. Furthermore, excessive heating in local areas at high power may also cause more thermal decomposition and thermoplastic deformation of the material, thereby intensifying the expansion of the microgroove width.

In addition, the formation of the deposited layers and their morphological features also underwent significant changes under different laser powers. All deposited layers exhibited a distinct LIPSS. At lower power settings (e.g., 15 mW and 20 mW), visible fragmentation and ejection of minute particles were observed at the periphery of the deposited layer, typically associated with the uneven heating of the material due to insufficient laser power to achieve complete melting. Under these conditions, the laser primarily induces surface microcracking, fragmentation, and particle ejection, rather than complete melting and vaporization, thus yielding a deposited layer with a discernible fragmented morphology. As the laser power increased to 25 mW, the deposited layers’ organization became increasingly homogeneous, and fragmentation virtually vanished. This phenomenon suggests that a higher laser power is adequate to melt and resolidify the NCD’s surface material, thereby generating a uniform deposited layer. At this juncture, the laser-ablated heating and evaporation processes are more comprehensive, with no evident fragmentation or particle ejection, contributing to a more consistent surface structure of the deposited layer. Nevertheless, upon further increasing the laser power to 30 mW and 35 mW, visible fragmentation re-emerged on the surface of the deposited layer. At these elevated power levels, the laser’s thermal impact on the NCD surface became excessively intense, potentially causing fragmentation or ejection of the molten material due to inadequate surface tension, ultimately adhering to the surface of the deposited layer. Consequently, while a higher laser power facilitates the melting and removal of surface material on NCD, excessive thermal action may lead to unstable deposited layer structures.

**Figure 5:**
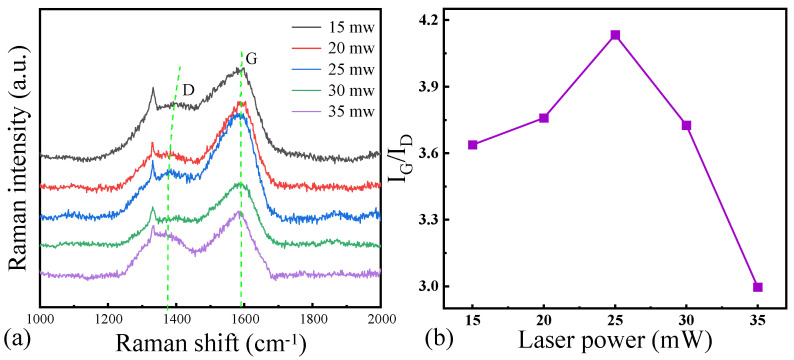
(**a**) Raman spectra and (**b**) l_G_/l_D_ ratio of the surface of NCD at different laser powers.

Figure 5a shows the results of the Raman spectroscopy on the surface of the laser-ablated microgrooves at different laser power levels, and Figure 5b shows the corresponding I_G_/l_D_ ratio in the Raman spectroscopy curve. The observations of the G peak and the D peak indicate varying degrees of graphitization on the NCD surface during laser ablation. Initially, when the laser power was in the range of 15 mW to 25 mW, the I_G_/I_D_ ratio increased progressively, indicating an enhancement in the degree of graphitization with increasing laser power. This phenomenon can be attributed to the effect of the laser power on the surface temperature of the NCD. A higher laser power resulted in more intense local heating, which facilitated the structural transformation of the diamond, leading to the partial disruption of the diamond crystal lattice and its conversion into graphite or graphitized products. However, when the laser power was further increased to 30 mW and 35 mW, the I_G_/I_D_ ratio exhibited a decreasing trend. This decline may be due to local overheating caused by excessive laser power, leading to the evaporation of graphite from the NCD surface or excessive lattice damage. The microstructural analysis of the deposited layer revealed that at a laser power of 25 mW, the deposited layer exhibited a relatively uniform and smooth morphology, suggesting that good surface uniformity enhances the degree of graphitization. A smooth surface typically indicates reduced laser debris generation and minimized local temperature fluctuations, which are conducive to a more stable graphitization process.

### 3.3. The Effect of the Defocus Level on the Graphitization of NCD

**Figure 6:**
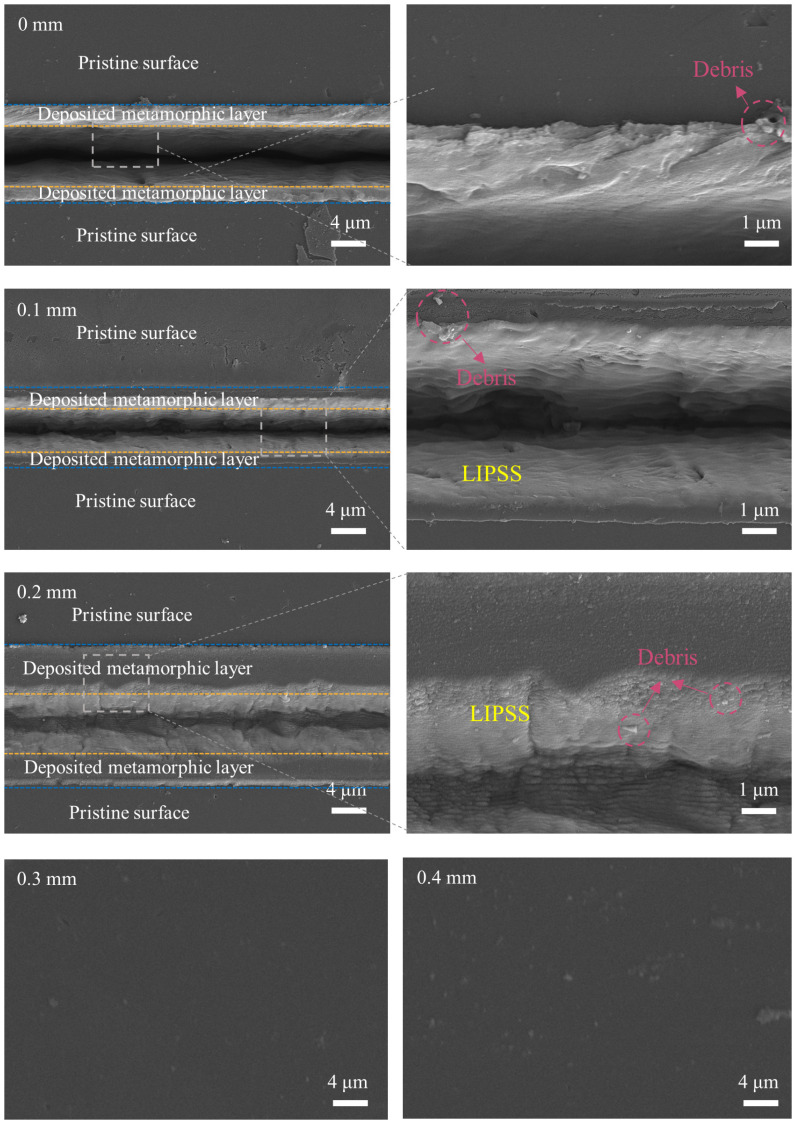
The influence of the defocus level on the morphology of the ablated NCD surface.

With the optimal laser scanning speed of 0.1 mm/s and laser power of 25 mW, subsequent nanosecond laser ablation of NCD was carried out to investigate the effect of the defocus level on the graphitization of NCD. The defocus level ranged from 0 mm to 0.4 mm, with an interval of 0.1 mm. For each defocus level, the other laser ablation parameters were the same, with a laser scanning speed of 0.1 mm/s and a laser power of 25 mW. Figure 6 illustrates the ablated surface morphology of the NCD at varying defocus levels. As the defocus level increased from 0 mm to 0.2 mm, a pronounced microgroove structure became evident on the NCD surface, with the depth of the microgrooves progressively diminishing. This phenomenon can be attributed to the change in the relative position between the laser’s focal point and the material surface. When the defocus level was zero, the laser beam was precisely focused on the NCD surface, concentrating the laser energy and causing localized high heat accumulation, which led to material evaporation or melting and the formation of microgrooves. As the defocus level increased, the focal point of the laser beam progressively shifted away from the NCD surface, causing the laser energy distribution to become more diffuse and spread out. This dispersion of energy reduced the local energy density and diminished the thermal effect on the NCD, resulting in a gradual reduction in the depth of the microgrooves. When the defocus level reached 0.3 mm and 0.4 mm, no significant microgrooves were observed on the surface, and there was no evident material removal from the NCD surface. At these defocus levels, the laser beam focus was far from the NCD surface, and the laser energy density was significantly reduced, leading to ineffective thermal interactions and an inability to remove material from the NCD surface. For defocus levels of 0 mm, 0.1 mm, and 0.2 mm, a noticeable deposited layer was observed on the NCD surface, exhibiting a linear periodic structure. At a defocus level of 0.2 mm, the area of the deposited layer on the NCD surface was maximized, and the bottom of the microgrooves also displayed LIPSSs. Further increases in the defocus level resulted in no deposited layer formation on the surface.

**Figure 7:**
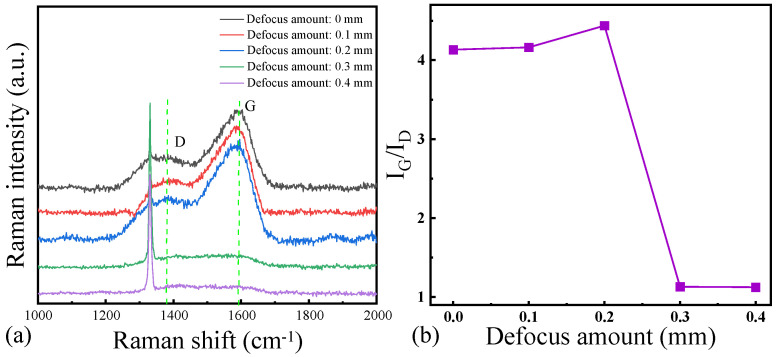
(**a**) Raman spectra and (**b**) l_G_/l_D_ ratio of the surface of NCD at different defocus levels.

Figure 7a shows the results of the Raman spectroscopy on the surface of the laser-ablated microgrooves at different defocus levels, and Figure 7b shows the corresponding IG/lD ratio in the Raman spectroscopy curve. The observations of the G peak and the D peak indicate varying degrees of graphitization on the NCD surface during laser ablation. First, at defocus levels of 0, 0.1, and 0.2 mm, the Raman spectra clearly exhibit the presence of both G and D peaks, indicating that under these conditions, laser ablation induces graphitization. The D peak is associated with the defect structure of graphitized regions, while the G peak corresponds to the vibrational modes of carbon–carbon bonds within the planar structure of the graphite. Consequently, the intensities of the G and D peaks serve as indicators of the extent of graphitization during laser ablation. When the defocus level increases to 0.3 mm and 0.4 mm, the intensities of the D and G peaks in the Raman spectra diminish, and in some instances, they become nearly indiscernible. This phenomenon can be attributed to the focusing effect of the laser energy. At lower defocus levels (0, 0.1, 0.2 mm), the laser beam is more tightly focused, resulting in higher energy densities that are sufficient to induce localized thermal decomposition and graphitization of the material. Conversely, at higher defocus levels (0.3 mm and 0.4 mm), the focusing of the laser beam becomes less precise, leading to a reduction in energy density and a consequent decrease in the localized heating effect, thus diminishing the extent of graphitization. The intensities of the D and G peaks also decrease correspondingly. By analyzing the I_G_/I_D_ ratio at various defocus levels, we can further assess the changes in the degree of graphitization. As the defocus level increases from 0 to 0.2 mm, the I_G_/I_D_ ratio gradually rises, suggesting an increase in the degree of graphitization on the material surface and a corresponding rise in the relative content of graphite. This trend is likely due to the enhanced ability of the laser beam to locally heat the material at lower defocus levels, thereby promoting graphitization reactions. However, when the defocus level reaches 0.3 mm and 0.4 mm, the I_G_/I_D_ ratio declines sharply, indicating a reduction in the graphitization process, which aligns with the weakened focusing effect and reduced energy density of the laser. 

### 3.4. The Effect of the Laser Scanning Interval on the Graphitization of NCD

In the surface laser scanning process, the selection and optimization of the laser scanning strategy, particularly the scanning interval, is of paramount importance. The laser linear scanning technique generally achieves localized melting or heat treatment by sequentially scanning discrete points along a predefined path on the material’s surface. In contrast, surface laser scanning necessitates that the laser beam covers the entire processing area, creating a continuous heating zone. Consequently, transitioning from line scanning to surface scanning involves not only the optimization of parameters for linear laser scanning but also the meticulous adjustment of the scanning interval to ensure the uniformity and efficacy of the surface treatment.

**Figure 8:**
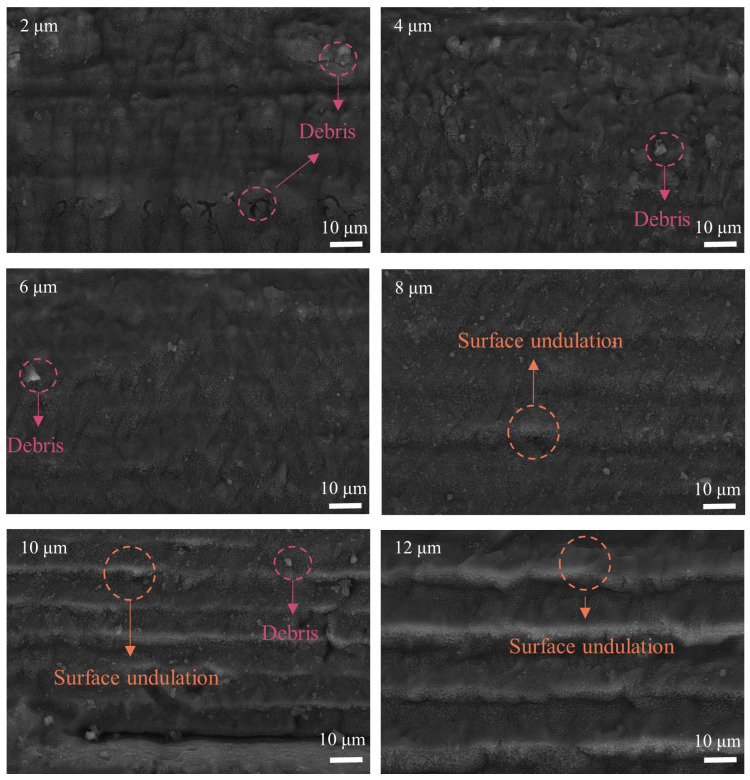
The influence of the scanning interval on the morphology of the ablated NCD surface.

With the optimal laser scanning speed of 0.1 mm/s, laser power of 25 mW, and defocus level of 0.2 mm, subsequent nanosecond laser ablation of NCD was carried out to investigate the effect of the laser scanning interval on the graphitization of NCD. The scanning interval ranged from 0 μm to 12 μm, with an increment of 2 μm. For each scanning interval, the other laser ablation parameters were the same, with a laser scanning speed of 0.1 mm/s, a laser power of 25 mW, and a defocus level of 0.2 mm. Figure 8 illustrates the ablated surface morphology of the NCD at varying scanning intervals. When the scanning interval is small (e.g., 2 μm, 4 μm, and 6 μm), the laser beam coverage is denser, and the overlap area of each laser pulse’s interaction with the material is minimal. While surface debris may still form, the overall surface remains relatively smooth. Specifically, at a 6 μm scanning interval, the modified surface exhibits a smoother finish, suggesting that the laser treatment is more uniform, and that localized melting is minimal. This promotes the formation of a more uniform graphitization layer and reduces surface defects. However, when the scanning interval increases to 8 μm, visible overlapping marks and small fragments become apparent on the surface. Further increasing the scanning interval (e.g., 10 μm and 12 μm) results in more pronounced overlapping marks, protrusions, and wavy structures. The increased scanning interval affects the thermal accumulation effect. A larger scanning interval implies a longer duration of laser beam exposure on the NCD surface, which can lead to excessive heat accumulation, causing non-uniform melting and vaporization, and thus forming more significant surface ripples or protrusions.

**Figure 9:**
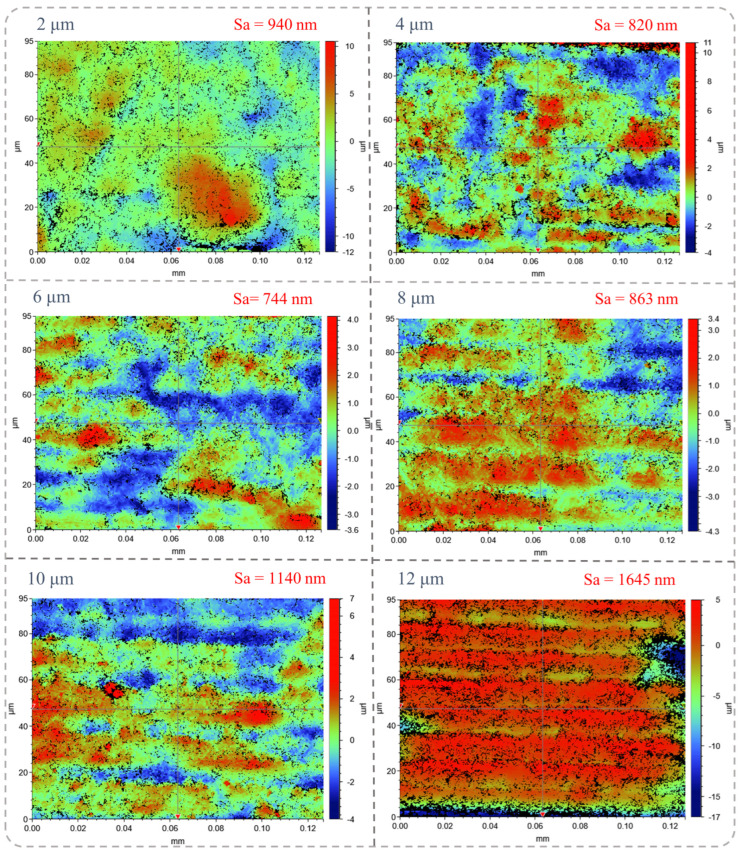
Three-dimensional surface morphology of laser-ablated NCD surface.

Figure 9 illustrates the three-dimensional surface morphology of the microgrooves on the laser ablation surface under varying scanning interval conditions. As shown in Figure 9, the surface roughness parameter Sa (arithmetic mean height deviation), obtained through white light interferometry characterization, serves as an ISO-standardized metric that effectively represents the overall undulation characteristics of the surface. Based on the observational findings, lower scanning intervals (e.g., 2 μm and 4 μm) led to an increased number of point-like protrusions on the surface, resulting in greater surface roughness. During the laser scanning process, the localized heat accumulation caused by the laser beam resulted in small melting regions, which subsequently formed protruding structures upon cooling. The smaller scanning interval concentrated the laser beam’s coverage area, intensifying the local heat effect and leading to more frequent small protrusions and surface irregularities, thus increasing the surface roughness. When the scanning interval was increased to 6 μm, the thermal effect of the laser became more uniform, resulting in a more consistent overall surface morphology. In this scenario, the surface roughness reached a minimum value of 744 nm. At a 6 μm scanning interval, the heat-affected zone between the laser pulses was relatively moderate, promoting a more uniform local melting and vaporization process, which helped mitigate surface irregularities caused by local overheating and yield a relatively smooth surface. However, when the scanning interval was excessively large (e.g., 8 μm, 10 μm, and 12 μm), the surface undulation became more pronounced, leading to a further deterioration in surface roughness. Under these larger scanning intervals, the local overheating of the NCD surface resulted in more intense melting, vaporization, and recrystallization, creating non-uniform heat processes that formed distinct surface wave ridges and protrusions, significantly increasing the roughness.

**Figure 10:**
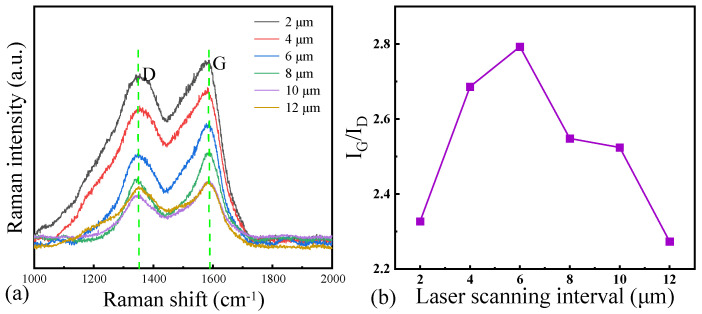
(**a**) Raman spectra and (**b**) l_G_/l_D_ ratio of the surface of NCD at different scanning intervals.

Figure 10a shows the results of the Raman spectroscopy on the surface of the laser-ablated surface of NCD at different scanning intervals, and Figure 10b shows the corresponding I_G_/l_D_ ratio in the Raman spectroscopy curve. As the scanning interval increases from 2 μm to 6 μm, a progressive rise in the I_G_/I_D_ ratio can be observed. The degree of graphitization reaches its peak when the scanning interval is set at 6 μm, resulting in the highest I_G_/I_D_ ratio. However, further increasing the scanning interval beyond 6 μm (e.g., to 8, 10, or 12 μm) leads to a decline in the I_G_/I_D_ ratio. At smaller intervals, structural inhomogeneities and lower graphitization levels are more prevalent due to greater overlap between laser beams and a less uniform energy distribution. As the interval increases, the reduced overlap and more even energy distribution promote a more uniform heat treatment, enhancing the overall graphitization process. However, excessively large intervals can result in significant thermal damage or incomplete coverage between laser beams, leading to surface irregularities and decreased graphitization.

## 4. Conclusions

In summary, we experimentally investigated the mechanism of surface graphitization during nanosecond laser ablation of NCD and its dependence on laser ablation parameters through systematic experimental research. The parameter optimization results obtained through a single-factor analysis require further validation via multivariate coupling studies. The rapid thermal excitation induced by the NCD absorbing laser energy resulted in elevated temperatures and pressures, leading to structural transformations where carbon atoms reconfigured into graphite lattice structures. Based on the analysis of the NCD surface morphology and degree of graphitization, it was determined that the single-factor-optimized parameters for achieving maximum graphitization without visible cracks or defects are 25 mW laser power, a scanning speed of 0.1 mm/s, and a defocus level of 0.2 mm. Additionally, based on laser linear scanning, optimizing the laser scanning interval to 6 μm can achieve the single-factor-optimized surface roughness and highest degree of graphitization for the modified surface. These findings provide valuable guidance for enhancing the performance of NCD-modified surfaces in microtexturing processes.

## Figures and Tables

**Table 1:** Laser ablation parameters of NCD.

Parameter	Range	Interval	Levels
Laser Power (mW)	15–35	5	5
Scanning Speed (mm/s)	0.05–0.25	0.05	5
Defocus (mm)	0–0.4	0.1	5
Scanning Interval (μm)	0–12	2	7

## Data Availability

The raw data supporting the conclusions of this article will be made available by the authors on request.

## References

[1] Tu J., Zhao Y., Chan S., Zheng Y., Chen L., Wei J., Liu J., Li C. (2024). Microstructure Control and Mechanical Properties of Ultra-Nanocrystalline Diamond Films. Ceram. Int..

[2] Lan J., Li H., Zhao X., Liu L., Li Y., Song H., Huang N. (2024). Investigation on Nanocrystalline Diamond Film with High Hardness. Vacuum.

[3] Katagiri K., Ozaki N., Umeda Y., Irifune T., Kamimura N., Miyanishi K., Sano T., Sekine T., Kodama R. (2020). Shock Response of Full Density Nanopolycrystalline Diamond. Phys. Rev. Lett..

[4] Ali B., Litvinyuk I.V., Rybachuk M. (2021). Femtosecond Laser Micromachining of Diamond: Current Research Status, Applications and Challenges. Carbon.

[5] Zhang J., Wang J., Zhang G., Huo Z., Huang Z., Wu L. (2024). A Review of Diamond Synthesis, Modification Technology, and Cutting Tool Application in Ultra-Precision Machining. Mater. Des..

[6] Xia Y., Lu Y., Yang G., Chen C., Hu X., Song H., Deng L., Wang Y., Yi J., Wang B. (2023). Application of Nano-Crystalline Diamond in Tribology. Materials.

[7] Sun F.H., Zhang Z.M., Shen H.S., Chen M. (2004). Growth of Nanocrystalline Diamond Films on Co-Cemented Tungsten Carbide Substrates by Hot Filament CVD. Mater. Sci. Forum.

[8] Liu H., Zong W., Cheng X. (2021). Behaviors of Carbon Atoms Induced by Friction in Mechanical Polishing of Diamond. Comput. Mater. Sci..

[9] Yan Y., Lei X., He Y. (2022). Experimental Study on the Effect of Surface Texture on Tribological Properties of Nanodiamond Films under Water Lubrication. Proc. Inst. Mech. Eng. Part J J. Eng. Tribol..

[10] Zhang Q., Wang C., Zhang H., Zhang S., Liu Z., Legut D., Veprek S., Zhang R. (2020). Designing Ultrahard Nanostructured Diamond through Internal Defects and Interface Engineering at Different Length Scales. Carbon.

[11] Chaudhri M.M. (2020). Indentation Hardness of Diamond Single Crystals, Nanopolycrystal, and Nanotwinned Diamonds: A Critical Review. Diamond Relat. Mater..

[12] Vincent C., Monteil G., Barrière T., Gelin J. (2008). Control of the Quality of Laser Surface Texturing. Microsyst. Technol..

[13] Wang H., Wen Q., Xu X., Lu J., Jiang F., Cui C. (2021). Ablation Characteristics and Material Removal Mechanisms of a Single-Crystal Diamond Processed by Nanosecond or Picosecond Lasers. Opt. Express.

[14] Calvani P., Bellucci A., Girolami M., Orlando S., Valentini V., Polini R., Mezzetti A., Di Fonzo F., Trucchi D.M. (2016). Infrared Absorption of Fs-Laser Textured CVD Diamond. Appl. Phys. A.

[15] Hazzan K.E., Pacella M., See T.L. (2021). Laser Processing of Hard and Ultra-Hard Materials for Micro-Machining and Surface Engineering Applications. Micromachines.

[16] Tan R., Yu Z., Li Y., Jiang H., Yu P., Xu J. Monitoring Method of Acoustic Emission Technology for Micro Texture of PCD Tool. Proceedings of the 2023 IEEE International Conference on Manipulation, Manufacturing and Measurement on the Nanoscale (3M-NANO).

[17] Mastellone M., Bolli E., Valentini V., Orlando S., Lettino A., Polini R., Buijnsters J.G., Bellucci A., Trucchi D.M. (2023). Surface Nanotexturing of Boron-Doped Diamond Films by Ultrashort Laser Pulses. Micromachines.

[18] Khomich A.A., Kononenko V., Kudryavtsev O., Zavedeev E., Khomich A.V. (2022). Raman Study of the Diamond to Graphite Transition Induced by the Single Femtosecond Laser Pulse on the (111) Face. Nanomaterials.

[19] Pimenov S., Zavedeev E., Arutyunyan N., Zilova O., Shupegin M., Jaeggi B., Neuenschwander B. (2017). Femtosecond-Laser Surface Modification and Micropatterning of Diamond-like Nanocomposite Films to Control Friction on the Micro and Macroscale. J. Appl. Phys..

[20] De Feudis M., Caricato A.P., Martino M., Ossi P., Maruccio P., Monteduro A.G., Corrado M. (2015). Realization and Characterization of Graphitic Contacts on Diamond by Means of Laser.

[21] Zhang Z., Zhang Q., Xu J. (2021). The Crack Propagation and Surface Formation Mechanism of Single Crystalline Diamond by a Nanosecond Pulsed Laser. J. Appl. Phys..

[22] Odake S., Ohfuji H., Okuchi T., Kagi H., Sumiya H., Irifune T. (2009). Pulsed Laser Processing of Nano-Polycrystalline Diamond: A Comparative Study with Single Crystal Diamond. Diamond Relat. Mater..

[23] Ohfuji H., Okuchi T., Odake S., Kagi H., Sumiya H., Irifune T. (2010). Micro-/Nanostructural Investigation of Laser-Cut Surfaces of Single- and Polycrystalline Diamonds. Diamond Relat. Mater..

[24] De Feudis M., Caricato A.P., Taurino A., Ossi P.M., Castiglioni C., Brambilla L., Maruccio G., Monteduro A.G., Broitman E., Chiodini G. (2017). Diamond Graphitization by Laser-Writing for All-Carbon Detector Applications. Diamond Relat. Mater..

[25] Ali B., Xu H., Chetty D., Sang R.T., Litvinyuk I.V., Rybachuk M. (2022). Laser-Induced Graphitization of Diamond under 30 Fs Laser Pulse Irradiation. J. Phys. Chem. Lett..

[26] Kononenko V.V., Gololobov V.M., Konov V.I. (2016). Latent Laser-Induced Graphitization of Diamond. Appl. Phys. A.

[27] Wang P., Zhang C., Jiang S., Duan X., Zhang H., Li L., Yang W., Liu Y., Li Y., Sun L. (2021). Density-Dependent Shock Hugoniot of Polycrystalline Diamond at Pressures Relevant to ICF. Matter Radiat. Extrem..

[28] Casiraghi C., Ferrari A.C., Robertson J. (2005). Raman Spectroscopy of Hydrogenated Amorphous Carbons. Phys. Rev. B.

[29] Yuan H., Zhao L., Zhang J. (2024). Experimental Parametric Investigation of Nanosecond Laser-Induced Surface Graphitization of Nano-Crystalline Diamond. Materials.

[30] Granados E., Martínez-Calderon M., Gomez-Aranzadi M., Rodríguez A., Olaizola S. (2017). Photonic Structures in Diamond Based on Femtosecond UV Laser Induced Periodic Surface Structuring (LIPSS). Opt. Express.

[31] Pimenov S.M., Zavedeev E.V., Arutyunyan N.R., Jaeggi B., Neuenschwander B. (2022). Femtosecond Laser-Induced Periodic Surface Structures on Diamond-like Nanocomposite Films. Diamond Relat. Mater..

